# Œdematous Laryngitis Successfully Treated by Scarifications of the Epiglottis, &c.

**Published:** 1852-03

**Authors:** 


*Six Cases of Œdematous Laryngitis, successfully treated by Scarifications of the Epiglottis and Aryteno-Epiglottic Folds.* Communicated by GURDON BUCK, Jr., M. D., Surgeon to the New York Hospital, &c.

The treatment of Œdema of the Glottis after the plan pursued so successfully by Dr. Buck, and by others at his suggestion, seems to us to form one of the most important of recent improvements in practical medicine. With a view to placing facts establishing the merits of the subject before those of our readers who have not access to the last volume of the Transactions of the Am. Med. Association, we copy from that work the following cases submitted in connection with the Report on Surgery. An account of previous cases may be found in the first volume of the Transactions.—EDITOR BUFFALO MED. JOURNAL.

In the first Volume of the Transactions of the American Medical Association, p. 137, a new method of treating this most formidable disease by scarifying the œdematous parts themselves, was made known to the profession, and its practical value shown by favorable results obtained from its employment in several cases.

Though this method originally occurred to the author as the result of his own reflections, his subsequent researches led him to the discovery that he had not been the first to propose and carry it into effect, but that he had been preceded by Lisfranc, who published (*Journ. Gen. de Medecine*, tome lxxxiii,) in 1823, a memoir on the subject, in which he stated that he had employed scarification in six cases. In five of them, an immediate change for the better took place, and resulted in complete recovery. In the sixth case, the operation, though repeated several times, only served as a palliative, death at length ensuing from extensive lesions of the larynx.

Strange to say, this important discovery of Lisfranc's fell into entire neglect, so much so, that Cruveilhier said of it, in 1834, (*Dict. de Med. et de Chir. Prat.* tome ii, p. 41,) "I doubt whether this little operation has ever been performed." Ryland also says of it (*Treatise on the Diseases and Injuries of the Larynx and Trachea*, Philad. 1838, p. 51,) "That it is fantastic, very difficult, if not impossible of accomplishment, and more likely to increase than diminish the existing mischief." Lisfranc's experience was, however, fully confirmed by the favorable results obtained in those cases reported by me in 1848 to this Association, all of which recovered promptly.

In two of them, no other efficient auxiliary treatment was employed. In two others, though venesection, leeches, emetics, mercury, &c., were also used, no substantial relief was obtained until scarifications were employed. In the fifth case, notwithstanding tracheotomy was resorted to after scarifications had been applied, there was reason to believe, as is set forth in the report alluded to, that scarification alone might have been relied upon.

Of the six cases about to be related, four came under my own personal observation during their progress. In all of them, the diagnosis of the disease was indubitable; and the fact that in every instance the operation was performed by other physicians, affords the most satisfactory corroborative testimony to the value and efficacy of this method.

Case I. Reported by Benjamin Vreeland, M. D., Resident Surgeon N. Y. Hospital, who performed the operation. Patrick Feeney, a laborer, aged twenty-six years, born in Ireland, was admitted into the New York Hospital, Ward No. 2, June 15th, 1849, at ten o'clock, A. M., with an extensive superficial burn of the face and arms, that happened a short time before, from an explosion of gas, occasioned by his going with a lamp into a cellar where a gas pipe was leaking. Numerous vesicles had formed upon the burnt surfaces; patient suffered but little pain, and appeared otherwise in good condition. He made no complaint of his mouth or throat even in swallowing, in consequence of which, these parts were not inspected at the time. Lint, smeared with a mixture of lime-water and linseed oil, was applied to the burns.

At six P. M., the following day, (June 16th,) the head, face, and arms, as also the neck anteriorly, and the upper part of the chest, which had been gradually swelling during the preceding twenty-four hours, had attained an enormous size, especially the face, which could hardly be recognized. The swelling was everywhere quiet, tense, and painful, but without œdema and without much change of color. Respiration was increased in frequency, and performed with difficulty, particularly inspiration, which was very short, and accompanied with a hoarse, croupy, metallic sound, while expiration remained quite natural. He complained a good deal of his throat; his voice was feeble and indistinct; he had no cough, the pulse was frequent and of good strength. Thirty leeches were applied to the throat, followed by hot fomentations. They bled freely, and afforded very considerable relief for a time, so that inspiration ceased to be attended with the peculiar sound noted above, and had become much easier. At about eleven o'clock the same evening, all the symptoms returned with aggravated violence, so much so, that it was with the utmost difficulty that the patient could breathe. Inspirations were much shorter, and often interrupted by choking paroxysms. On introducing the finger into the throat, the epiglottis was felt to be quite natural, but on either side a large firm swelling was perceived rising up into the pharynx, and encroaching upon the entrance of the larynx. The whole inside of the mouth appeared to be also involved in the general swelling, but without any abrasion or ulceration.

Scarifications were immediately determined upon and performed in the manner of, and with the instrument recommended by, Dr. Buck. Patient, being sensible of his desperate condition, maintained his mouth wide open, with remarkable resolution, and thus rendered the operation easy of accomplishment. The instrument was introduced, two separate times, at an interval of a few moments, and the swollen parts freely scarified. Two or three ounces of blood mixed with sputa were hawked up, and patient expressed immediate relief.

Inspiration became easy, diminished in frequency, and ceased to be noisy.

*June* 18*th.* Patient's progress has continued favorable, without any recurrence of dyspnœa. The parts about the epiglottis have resumed their natural size and condition. The swelling of the face is beginning to subside. On the 1st of July, patient was sitting up, and convalescing rapidly.

Subsequently, on July 13th, 1849, he was discharged cured.

CASE II. Reported by Frederick D. Lente, M. D., Resident Surgeon in the New York Hospital, who performed the operation. Chas. Foley, waiter, ætat. twenty years, born in Ireland, was sent to the Hospital May 2d, 1850, by his medical attendant, for the purpose of having the operation of tracheotomy performed; the treatment that had been already employed having failed to relieve the urgent dyspœna from which he suffered, and which depended on disease of the larynx. Patient was emaciated, blanched, and feeble, and presented both the rational and physical signs of phthisis, such as cough, night-sweats, progressive loss of flesh and strength, &c., with dullness on percussion under the left clavicle, and prolonged expiration. His countenance was anxious, his breathing difficult, and accompanied with occasional paroxysms of suffocation, which had become more violent the night previous to his admission. Deglutition was very difficult, and his voice reduced to a whisper. Pressure about the larynx gave him pain. The finger passed down the throat perceived the epiglottis in a natural condition. The fauces and tonsils were red and a little swollen. Patient's attack had commenced with sore throat a week ago, after exposure to dampness. Two days ago, the sore throat still continuing unabated, difficulty of breathing had supervened, and had gone on increasing till the time of his admission. The tongue was furrowed, pulse frequent and feeble, surface rather cool. Besides other treatment, patient had been kept nauseated with tartar emetic. Treatment after admission: ten grains of calomel were given, and followed by oil. A tobacco poultice was applied to the throat, and mustard pediluvium resorted to. As bedtime approached, without any relief of the dyspnœa, Doctor Lente applied to the interior of the larynx a strong solution of nitrate of silver by means of a probang. Instantly a convulsive paroxysm of suffocation was produced, and for a few seconds threatened the life of the patient. After waiting a minute or two, and finding the danger of suffocation still continuing imminent, Dr. Lente summoned Dr. Vreeland, the other resident surgeon, to his assistance, and proceeded to scarify the epiglottis and aryteno-epiglottic folds; the first with a guarded curved bistoury, the latter with Dr. Buck's scarifying knife. Patient was almost immediately relieved, and within five minutes could speak in an articulate voice, and swallow fluids readily. Patient progressively improved without any repetition of the operation, and on the 13th of the month (May) was discharged, cured of the laryngeal affection.

CASE III. Operation by Alfred C. Post, M. D., Surgeon to New York Hospital.

George Burnett, seaman, æt. twenty-seven, born in New Hampshire, of robust constitution, was admitted into Ward No. 12, second Surgical Division, with œdema of the glottis, that had supervened in the progress of deep inflammation of the cellular tissue of the neck. On the preceding Wednesday, after returning home from his work, patient was unable to take his supper, owing to soreness of the throat. On the following day, his neck began to swell, and on Friday dyspnœa commenced, and gradually increased to such a degree that the patient became alarmed, and sought relief at the Hospital on Tuesday forenoon, June 4th, 1850, when his condition was as follows:

The anterior part of the neck, from the base of the jaw to the top of the sternum, and clavicles, was so much swollen as to be advanced forward to a line with the chin. The skin and subjacent tissues were consolidated together by adhesive inflammation. The surface was red, indurated, and shining.

The outlines of the muscles and other constituent parts could no longer be identified by the sight or touch. The lower jaw could only be depressed to about one-half its usual extent. Dyspnœa had become very urgent. Inspiration was performed with a good deal of effort, and attended, though not constantly, with a hoarse croupy sound, while expiration was easy. The voice could not be raised above a whisper; attempts to swallow caused a good deal of disturbance and aggravated dyspnœa. No distinct paroxysms of suffocation had occurred except when excited by attempts to swallow. Patient was unable to assume the recumbent posture. On depressing the tongue, the epiglottis could be seen swollen, erect and folded upon itself. The aryteno-epiglottic folds could be reached with the finger, and felt to be swollen and pulpy, as well as the epiglottis.

At two o'clock, P. M., Dr. Post, being in attendance during my absence, scarified the epiglottis and aryteno-epiglottic folds with a sharp-pointed curved bistoury, guarded by a strip of adhesive plaster wound around the blade to within half an inch of the point, the tongue being depressed at the same time with a spatula. The scarifications bled pretty freely, and patient experienced prompt relief. Twelve leeches were then applied externally over the larynx.

At six o'clock the same afternoon, the patient came under my own charge. The relief afforded by the scarifications still continued; his voice had nearly resumed its natural tone; his pulse was ninety-eight, strong and full; ordered one-fourth of a grain of tartar emetic in solution every two hours, and a blister to the nape of the neck.

*5th.* Had passed a comfortable night, and continued to do well; had vomited several times. The dyspnœa having somewhat increased, a second application of twelve leeches was made over the larynx at eight o'clock, A. M. The epiglottis still continued swollen, but to a less extent. Deglutition has become somewhat easier. Pulse 108. Bowels costive; ordered comp. infus. senna; poultice to be continued to the throat.

*6th.* Found patient walking about the ward; he had slept well; swallows fluids with facility. Epiglottis in the same condition. Tartar emetic ordered at longer intervals.

*8th.* Patient continues to improve, and is no longer obliged to have his head raised when lying down. The external swelling of the neck has diminished; the redness of the surface has for the most part disappeared, and the skin has begun to resume its natural softness; the subcutaneous tissues are still hard and tense, especially below the os hyoides, and as far down as the sternum. The right half of the epiglottis is still swollen and red, and its margin appears ulcerated. The left half has nearly resumed its natural size and appearance. The voice remains husky, and a little hoarse cough is at times troublesome. Ordered oxymel scillæ ʒi every two hours; emplastrum saponis to the throat.

*10th.* Still improving; ordered camphorated mercurial ointment to be rubbed in over the throat morning and evening, and oiled silk to be kept applied to it.

*15th.* The epiglottis is natural. Cough has disappeared; neck continues improving.

*22d.* Improvement of the neck has progressed rapidly since the above date.

*25th.* Patient was discharged cured.

Case IV. Operation by Alfred C. Post, M. D., Surgeon to the New York Hospital.

Robert Whitten, boatman, æt. twenty-five, born in Pennsylvania, of good constitution, was admitted August 21st, 1850, into Ward No. 8, second Surgical Division, New York Hospital, with gonorrhœal orchitis, and had so far recovered as to be about the premises, when he was seized, on the 8th October, while at dinner, with extreme difficulty of swallowing, and darting pains, radiating from the throat to the left ear, which obliged him to take his bed. Patient had had soreness of his throat for five or six days previous, which he attributed to exposure near an open window in the night, and which he had supposed was gradually getting better, till the moment of the attack just noticed. During the night of the 8th, he became much worse, and, on the following morning, the 9th, his breathing was very difficult and stridulous, with occasional paroxysms of increased dyspnœa, which were always brought on by attempts to swallow. The voice was reduced to a whisper, and great effort was required to use it. Deglutition was also very painful. On inspection, the uvula was found elongated and œdematous; the epiglottis was folded upon itself, and very much swollen from serous effusion into the submucous cellular tissue covering its lingual surface. To the touch, the aryteno-epiglottic folds, which were also swollen, had a pulpy feel, as well as the epiglottis. The fauces and tonsils were red, and moderately swollen; the pulse was full and frequent. At one o'clock, Dr. Post, finding the patient in the above condition, scarified the œdematous epiglottic and aryteno-epiglottic folds with the scarifying knife, guided upon the forefinger of the left hand previously introduced, so as to fix the epiglottis against the base of the tongue. As soon as the patient could recover sufficiently from the strangulating effects of the operation, he exclaimed, "Doctor, you will kill me!" The relief afforded was immediate and most decided; considerable blood was hawked up with the sputa, after the operation.

Eight leeches were applied, and followed by poultices; one-fourth of a grain of tartar emetic in solution was ordered to be taken every two hours.

*October 8th*, morning. Patient was much better, with very little dyspnœa remaining; his voice continued good; the inflammation of the fauces and tonsils still continued, though somewhat abated; antimony ordered to be continued.

*12th.* Improvement satisfactory; ordered Bell's gargle.

*15th.* A solution of nitrate of silver, forty gr. to ℥i, has been applied daily, for three days past, to the fauces; patient goes about the house.

*21st.* Patient is well, epiglottis and fauces of normal appearance. Discharged cured, Oct. 22d, 1850.

Case V. Under Dr. A. C. Post's care. Operation by Dr. F. D. Lente, Resident Surgeon N. Y. Hospital.

John Burriss, seaman, æt. twenty-five years, born in Maine, was admitted into Ward No. 9, second Surgical Division, Nov. 16th, 1850, at noon, with suppurating glandular swellings of the neck, which first appeared about eighteen months previously; the opening had not, however, formed till within six weeks. During the three days immediately preceding his entrance into the hospital, he had been very much exposed to cold and wet, on a journey from Quebec to this city.

In consequence of this exposure, patient became worse, and presented the following condition at the time of his admission: The right side of the neck was swollen, especially under the angle of the jaw and along the upper half of the sterno-mastoid muscle, where several prominences indicated the existence of large lymphatic glands. To some distance around, the skin and subjacent cellular tissue were thickened, indurated, and tense; the surface was red and shining. From three or four ulcerated openings, a purulent discharge was taking place. These openings, as well as the cicatrices, of other openings that had recently closed, presented a well-marked scrofulous aspect. Deglutition was difficult, and respiration somewhat labored; the voice was also hoarse. At six the same evening, patient was suddenly seized with a paroxysm of dyspnœa, threatening instant suffocation, and obliging him to start up from his bed for relief. The nurse, in great alarm, ran for Dr. Lente, the house surgeon, who attended promptly, and found the patient sitting on the side of his bed, in the greatest distress from extreme difficulty of breathing, depending on obstruction of the larynx. His face was livid, and his voice reduced to a whisper. Dr. Lente's attention was first directed to the external swellings of the neck, and, finding a fluctuating point, punctured it; a small quantity of matter only was discharged, but without any relief. The rigidity of the lower jaw preventing an inspection of the fauces, Dr. Lente introduced his forefinger into the mouth and explored the epiglottis, which was found swollen, erect, folded upon itself, and as large as the end of his thumb. Dr. Church, the resident surgeon of the First Division, confirmed, by examination, what Dr. Lente had observed.

Dr. Lente first scarified the epiglottis with a sharp-pointed curved bistoury, guarded to within half an inch of its point by a narrow strip of adhesive plaster wound around the blade. He then scarified the aryteno-epiglottic folds with the scarifying knife used for this purpose, which he conducted along the dorsum of the left forefinger first introduced into the mouth, so as to overlap the epiglottis, and press it forwards against the tongue.

After clearing the fauces of blood and sputa, patient expressed himself greatly relieved, and before the doctor left him he was able to speak in a loud tone, and to swallow with greater facility than he had done for three days. One dozen leeches were applied to the throat, and followed by tobacco poultice. Ten grains calomel were also given.

*November 17th*, morning. Patient was much better; the epiglottis had already resumed its natural size; ordered comp. infus. sennæ.

19*th*. Improvement continued uninterruptedly; the voice had become natural; patient remained under treatment for the glandular swellings of his neck until the 30th of December, but there was no return of the laryngeal affection.

Case VI. Occurred at the Bellevue Hospital during the attendance of Drs. Alonzo Clark and Samuel C. Foster, successively. The operation was performed by Dr. Leroy Ravenhill, house physician, to whose kindness I am indebted for this report.

James Conner, æt. forty-five, native of Ireland, and laborer by occupation, was admitted to the hospital March 14th, 1851. States that his previous health had been good, and habits temperate. His illness commenced a week previous to admission, with debility, headache, loss of appetite, and some obscure and wandering pains, but no well marked rigors.

On admission, his symptoms were those of a mild case of typhus fever, from which he soon began to recover, and continued to improve until the 24th, when he complained of soreness of the throat. On examination, no unnatural redness of the fauces was discovered.

Being the same on the following day, a mild astringent gargle was used, and fomentations externally. He continued much in the same condition the two succeeding days, a turpentine epithem having, in the mean time, been applied to the neck. When seen by the attendant, in the afternoon of the 28th, the dysphagia had increased, and there was a diffused tumefaction of the neck on either side, with tenderness on pressure. His voice, which had been slightly hoarse a day or two previous, was now quite muffled. Apprehensive of œdema of the glottis, particular inquiry was made in reference to his respiration. It was found to be but slightly accelerated, and he denied having any difficulty in breathing either in inspiration or expiration. The turpentine was reapplied. In the evening of the same day, he was observed, by the orderly of the ward, suddenly to rise up in bed and then fall back, apparently in great distress. Dr. Ravenhill, the house physician, was immediately called. He found him in an alarming condition; his limbs and body extended; his head thrown back; his face and lips purple, and covered with a cold sweat. Instead of replying to any question, he answered by signs, and kept putting his hand on the larynx as the seat of distress. Inspiration was exceedingly laborious, being accompanied with a slight croupal sound, and by manifest convulsive action of the respiratory muscles. Expiration was comparatively easy. The pulse had not varied since the last visit.

The finger, being introduced into the mouth, was carried back to the epiglottis, which felt natural at the apex, but somewhat tumefied at the base. Behind this tumefied part, two tense smooth swellings were found, which completely obstructed the entrance of the larynx, and thus prevented the admission of air. They could be separated a little, which allowed the patient to inspire with comparative ease. After some twenty inspirations were taken in this way, a hernia knife was procured, and directed carefully by the finger until its cutting portion had reached the left tumefaction, which was incised, and then the opposite swelling. The incisions were followed by immediate coughing, and expectoration of blood and frothy mucous, which lasted for about ten or fifteen minutes. The patient then began to breathe naturally, and to speak. The articulation was hoarse and gruff, but distinct. He was asleep within half an hour, and when seen about midnight, was still sleeping and breathing freely.

*29th*, A. M. Patient feels refreshed after the night's rest; eyes somewhat suffused; face natural in color; breathing free; deglutition somewhat difficult; voice not natural, rather husky; tongue slightly furred in the center; slight soreness experienced by pressing on the larynx; pulse eighty.

P. M. Symptoms the same as at the morning visit, except the voice, which seems more natural.

*April 1st.* Patient continues doing well; no longer any trouble in swallowing; voice still harsh; pulse seventy-six; tongue slightly furred; bowels costive; ordered ol. ricini. ℥ss.

Patient recovered completely from the ailment of his throat and larynx.

Deafness of the right ear, and a mild attack of erysipelas of the forearm, detained him in the hospital till the 25th of April, during the latter part of which time he acted as assistant to the orderly of the ward.

